# Combining a Risk Factor Score Designed From Electronic Health Records With a Digital Cytology Image Scoring System to Improve Bladder Cancer Detection: Proof-of-Concept Study

**DOI:** 10.2196/56946

**Published:** 2025-01-22

**Authors:** Sandie Cabon, Sarra Brihi, Riadh Fezzani, Morgane Pierre-Jean, Marc Cuggia, Guillaume Bouzillé

**Affiliations:** 1 Univ Rennes, CHU Rennes, INSERM, LTSI - UMR 1099 F-35000 Rennes France; 2 R&D VitaDX International Paris France

**Keywords:** bladder cancer, clinical data reuse, multimodal data fusion, clinical decision support, machine learning, risk factors, electronic health records, detection, mortality, therapeutic intervention, diagnostic tools, digital cytology, image-based model, clinical data, algorithms, patient, biological information

## Abstract

**Background:**

To reduce the mortality related to bladder cancer, efforts need to be concentrated on early detection of the disease for more effective therapeutic intervention. Strong risk factors (eg, smoking status, age, professional exposure) have been identified, and some diagnostic tools (eg, by way of cystoscopy) have been proposed. However, to date, no fully satisfactory (noninvasive, inexpensive, high-performance) solution for widespread deployment has been proposed. Some new models based on cytology image classification were recently developed and bring good perspectives, but there are still avenues to explore to improve their performance.

**Objective:**

Our team aimed to evaluate the benefit of combining the reuse of massive clinical data to build a risk factor model and a digital cytology image–based model (VisioCyt) for bladder cancer detection.

**Methods:**

The first step relied on designing a predictive model based on clinical data (ie, risk factors identified in the literature) extracted from the clinical data warehouse of the Rennes Hospital and machine learning algorithms (logistic regression, random forest, and support vector machine). It provides a score corresponding to the risk of developing bladder cancer based on the patient’s clinical profile. Second, we investigated 3 strategies (logistic regression, decision tree, and a custom strategy based on score interpretation) to combine the model’s score with the score from an image-based model to produce a robust bladder cancer scoring system.

**Results:**

We collected 2 data sets. The first set, including clinical data for 5422 patients extracted from the clinical data warehouse, was used to design the risk factor–based model. The second set was used to measure the models’ performances and was composed of data for 620 patients from a clinical trial for which cytology images and clinicobiological features were collected. With this second data set, the combination of both models obtained areas under the curve of 0.82 on the training set and 0.83 on the test set, demonstrating the value of combining risk factor–based and image-based models. This combination offers a higher associated risk of cancer than VisioCyt alone for all classes, especially for low-grade bladder cancer.

**Conclusions:**

These results demonstrate the value of combining clinical and biological information, especially to improve detection of low-grade bladder cancer. Some improvements will need to be made to the automatic extraction of clinical features to make the risk factor–based model more robust. However, as of now, the results support the assumption that this type of approach will be of benefit to patients.

## Introduction

Bladder cancer ranks as the 9th most prevalent cancer worldwide, and it is the 13th leading cause of cancer-related deaths, claiming over 13,000 lives annually [1,2]. In 2017, bladder cancer was the 5th most frequently diagnosed cancer in men, with an incidence 4 times higher than that in women [3]. Detecting bladder cancer at an early stage significantly enhances the chance of successful treatment, while late-stage detection presents more challenges. Current treatments involve surgical procedures and auxiliary chemotherapy but come with a relatively poor 5-year prognosis [4]. More precisely, the 5-year survival is 69.5% for localized disease. The survival rate decreases to 36.3% for regional disease and only 5% for metastatic bladder cancer [5]. Today, periodic cystoscopy associated with urine cytology is the gold standard for the diagnosis of bladder cancer or to monitor for recurrence [6]. However, cystoscopy is an expensive and invasive procedure usually associated with discomfort. Urine cytology is a test to detect abnormal cells in urine samples. Although urine cytology is the most widely used noninvasive test, it has poor sensitivity, especially for low-grade tumors [7,8].

Thus, to reduce the mortality associated with bladder cancer, new diagnostic tools need to be proposed for early detection of the disease [3]. At the same time, newly proposed methods should be noninvasive and inexpensive to allow large-scale dissemination. Research is currently focusing on this objective through better identification of risk factors or development of more effective diagnostic tools.

Recently, new diagnostic tools based on the detection of specific biomarkers in urine tests were proposed [9-11]. ADXBLADDER is a urine test to detect the mini chromosome maintenance 5 protein in voided urine [9]. It has a sensitivity of 83% and specificity of 77%. Urodiag is based on genetic and epigenetic analysis using multiplex quantitative real-time polymerase chain reaction and consists of detecting 4 mutations of the fibroblast growth factor receptor 3 gene [10]. It has a sensitivity of 95% and specificity of 76%. URO17 is an immunocytochemical test designed to evaluate keratine 17 expression and has a sensitivity of 100% and specificity of 96% [11]. These tests show promising performance but have only been studied in small cohorts (from 26 to 71 patients).

Increasing interest in deep learning algorithms has also led to the emergence of new diagnostic strategies based on image processing (see [12,13] for reviews). Although most of them have been developed from cystoscopy images, some aim to propose noninvasive techniques and have exploited images obtained from urine cytology [14-16]. The authors report good to very good performance, including a sensitivity of 79.5% and specificity of 84.5% [14]; area under the receiver operating characteristic (ROC) curve (AUC) of 0.83 [15]; and sensitivity of 81.0% and specificity of 98.0% [16]. However, studies were conducted either with populations composed mainly of patients with high-grade cancer [14] or with a small amount of data and a limited number of patients (ie, only 398 slides in [15] and 68 patients in [16]). Moreover, a lack of generalization power, as expressed by a decrease in performance when applying the model to new data and especially new data sources, hinders deployment in clinics [13]. In addition, the main challenge is detecting patients with low-grade cancer to improve their chance of survival.

On the other hand, several risk factors were identified as strong determinants of bladder cancer. First, demographic information, such as age and sex, was identified as a strong factor [5]. In 2012, Letašiová et al [17] also highlighted that smoking; exposure to arsenic (in water, air, or food); and exposure to aromatic amines and 4,4'-methylenebis in chemicals that are typically contained in dyes, hair dyes, paints, fungicides, cigarette smoke, plastics, metals, and motor vehicle exhaust are the most notable environmental risk factors [17]. An association with diabetes has also been described [18]. In addition, positive associations between bladder cancer and a family history in first- and second-degree relatives [19] and hypertension [20] were found. In a more general context, having experienced stroke [21] or heart failure [22] was also associated with a higher risk of developing cancer.

The widespread adoption of electronic health records now provides an opportunity to capture granular patient information in large populations. The data can be used to develop machine learning models that could be incorporated in decision support systems. By incorporating risk factors, such models have the potential to identify at-risk patients more accurately, enhancing patient profiles for image-based analysis.

In this paper, we proposed combining knowledge about risk factors and cytology processing software to investigate the benefits that this could provide for bladder cancer detection. To do this, our team worked with a model developed by the company VitaDX [23]. This image-based model, called VisioCyt, integrates image cell processing and deep learning algorithms to predict bladder cancer from voided urinary cytology. In this study, our focus was on building a detection model based on risk factors extracted from our local clinical data warehouse (CDW). We hypothesized that a model trained on a large data set, offering a probability of bladder cancer based on a patient's clinical profile, can complement the VisioCyt scoring system and enhance detection, especially for low-grade cases.

## Methods

### Ethical Considerations

Our study followed the relevant guidelines and regulations, in accordance with the Declaration of Helsinki. The extracted data did not contain any nominative data, and all information was deidentified in accordance with the protocol established for the improved project. Agreement from the French Commission Nationale de l’Informatique et des Libertés was obtained (agreement number 2206739).

### Study Design

**Figure 1:**
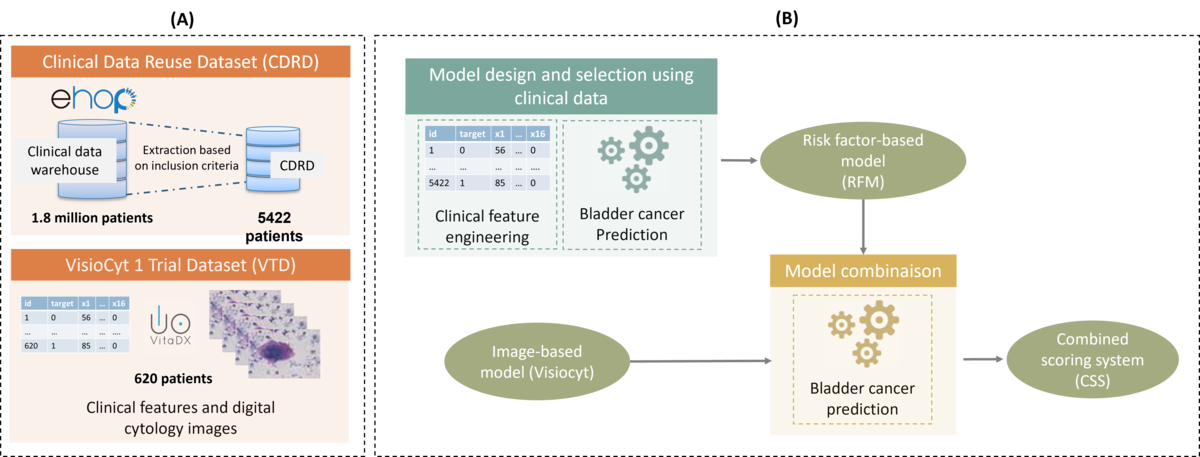
Overview of the designed workflow for the scoring of bladder cancer using risk factors and cytology images, including a description of the (A) data sets and (B) data processing.

The proposed workflow for bladder cancer prediction is depicted in Figure 1. As a first step, 2 distinct prediction models were built: one based on risk factors (risk factor–based model [RFM]) and the other using cytology images (VisioCyt). The scores given by each model were then combined to provide a composite score.

To set up and evaluate our approach, 2 data sets were considered: Clinical Data Reuse Dataset (CDRD) and VisioCyt 1 Trial Dataset (VTD).

CDRD was extracted according to the inclusion criteria from eHOP [24], the CDW developed and deployed at Rennes University Hospital. This database was used to design and select the bladder cancer prediction model based on risk factors (ie, the RFM). For that purpose, clinical features were retrieved and preprocessed to be used as inputs for the machine learning approaches. In the end, several models were compared to identify the best model.

The second data set contained clinical data and cytology images that were acquired during the VisioCyt 1 Trial (NCT02966691). Images from this data set were first used to set up the bladder cancer prediction model based on cytology images and deep learning (ie, VisioCyt). This model was internally developed by VitaDX. In this study, we focused on the combination of this model with an RFM. To evaluate and adopt the best strategy for the combination, the whole data set (ie, images and clinical data from VTD) was used.

In the following sections, each stage of this workflow is described. First, we introduce the study population with the associated inclusion criteria. It is followed by a description of the data sets. Methods to set up the risk factor–based predictor are presented, and a description of the Visiocyt model is given. Finally, the combination strategies to build the final bladder cancer predictor are described.

### Study Population

The eligibility criteria were defined in the VisioCyt 1 Trial [23] and used to extract data from the CDW.

The inclusion criteria were the following: older than 18 years, affiliation with a social security system, negative urine culture, programmed bladder endoscopy for a suspicion of bladder cancer (de novo, monitoring, or relapse) or exploration of the lower urinary tract excluding a suspected bladder cancer or prostate cancer.

The exclusion criteria were as follows: ongoing, untreated urinary tract infection; presence of bladder cancer excluding urothelial carcinoma; carcinoma of the high urinary tract, associated; history of lithiasis pathology; had undergone a renal transplantation.

### Data Sets

#### Clinical Data Reuse Data Set

With eHOP, administrative and clinical data from electronic health records are collected, including unstructured (eg, clinical notes) and structured (eg, drugs, laboratory results) data. Data are deidentified, and a unique anonymous identifier allows the linkage among hospital stays of a given patient. The eHOP CDW currently allows users to search 80 million unstructured data elements and 430 million structured elements. All these data cover more than 1.8 million patients. CDRD was extracted from the eHOP database. All data on hospital stays at Rennes University Hospital between 2015 and 2017 were considered.

In this study, we considered people who had undergone endoscopy for vesical tumor resection or had a histology examination that was positive for bladder cancer. People considered negative for bladder cancer were those who had undergone a bladder endoscopy and had a negative histological examination.

For this extraction, we used the following data: demographic data; drugs administered (codification used by the French hospital information system to identify a drug and date of administration); Programme de Médicalisation des Systèmes d'Information (PMSI) data (French diagnosis-related groups) including International Statistical Classification of Diseases, Tenth Revision (ICD-10) codes, procedure codes, mortality, and length of stay; laboratory results described with local terminology; medical reports.

The extraction resulted in 5422 eligible patients: 1351 bladder cancer positives and 4071 bladder cancer negatives.

#### VisioCyt 1 Trial Data Set

The VisioCyt 1 Trial is registered in clinicaltrials.gov (NCT02966691). This clinical study was designed to evaluate the diagnostic performance of the VisioCyt test, which would improve the early diagnosis of bladder cancer. The diagnostic method used by the VisioCyt device is based on the analysis by transmission of urinary cytology slides prepared according to the protocol outlined for VisioCyt [23].

In addition to the inclusion criteria presented in the Study Population section, other criteria specific to that trial were considered. Additional inclusion criteria were the ability of the patient to understand the protocol and signature of a consent form before beginning the study, while people deprived of liberty or under guardianship were excluded. In addition, patients included in the same hospital as that from which CDRD was extracted were also excluded to avoid double inclusion.

In VTD, patients were considered to have bladder cancer if they had positive urine cytology and positive histology. This resulted in 620 patients, that is, 409 bladder cancer positives and 211 bladder cancer negatives. Among the positive patients, 220 patients with low-grade cancer and 189 patients with high-grade cancer were included. We collected 2 types of data. First, VisioCyt 1 provides urinary cytology digital slide images at a size of 50,000 × 50,000 pixels. They were acquired over 3 focal plans and contain cells of different types (eg, malpighian cells, urothelial cells, neutrophilic polynucleosis, artifacts, blood cells). For each VTD patient, the following demographic variables were retrieved: age, sex, weight (kg), and height (cm). The following risk factors and comorbidities were also collected: smoking status (never, former, or current smoker), diabetes, history of heart failure, hypertension, history of stroke, chronic obstructive pulmonary disease (COPD), family history of cancer, and professional exposure to a carcinogenic factor.

### Risk Factor–Based Model (RFM)

#### Clinical Feature Extraction and Preprocessing

For each CDRD patient, the same features as those collected for VTD patients were retrieved. They were extracted from the data warehouse using structured and unstructured data. For structured data, standardized codes of several terminologies and local codes were used. For unstructured data (ie, medical reports), regular expressions and some more advanced techniques based on embeddings (ie, using spacy modeling from python) were implemented or used [25].

Missing data imputation was performed for weight and height by using k-nearest neighbor identification (k=10) based on age, sex, and weight or height according to the feature to be imputed. Standard scaling was first applied to ensure an equivalent impact on the distance measurement for numerical variables. For binary variables, we assumed that patients did not have the feature if it was not retrieved through the queries used on the data warehouse. A BMI feature was added and computed as weight divided by height squared.

A missing data indicator feature was added for each of the original features except for age and sex, which were complete. In the end, the clinical feature set had 29 clinical features.

#### Bladder Cancer Prediction From Clinical Data

We investigated 3 supervised classification algorithms, namely random forest (RF), logistic regression (LR), and support vector machine (SVM), to identify patients likely to have bladder cancer based on their clinical data.

**Figure 2:**
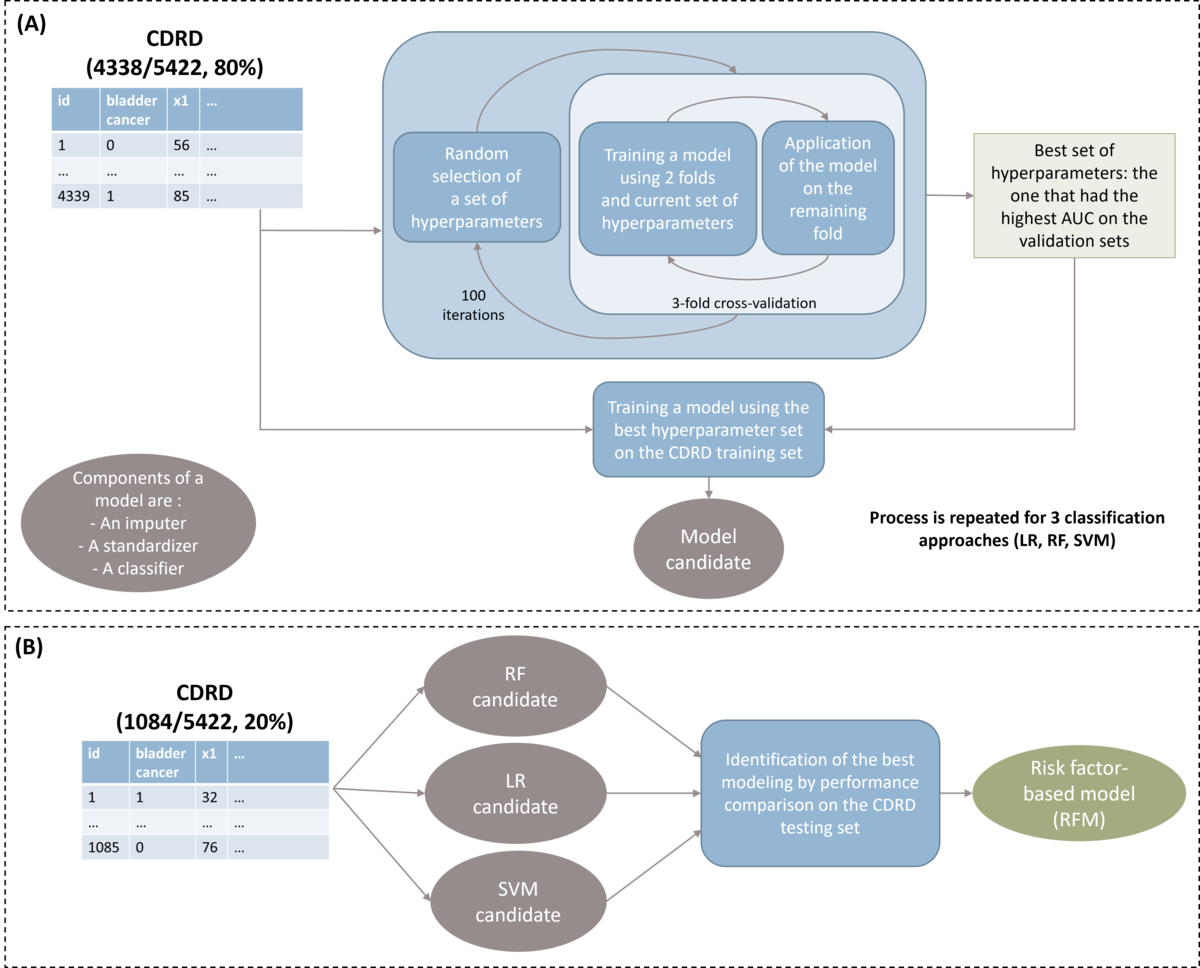
Identification of the best classification model using 2 steps: (A) The first was training, a selection of the best set of hyperparameters and fit of a model candidate to use for each classification approach, and (B) the second was testing, identification of the best model among the 3 candidates. AUC: area under the receiver operating characteristic curve; CDRD: Clinical Data Reuse Dataset; LR: logistic regression; RF: random forest; SVM: support vector machine.

A 2-step strategy, depicted in Figure 2, was designed to identify the best modeling approach.

First, the most optimal hyperparameters were identified using 3-fold cross-validation and a random grid search on 80% of CDRD (100 iterations). The best set of hyperparameters was defined as the one leading to the highest averaged AUC on the validation sets. During training, class imbalance was handled using class-weighting to mitigate the risk of bias toward the majority class in splits (ie, no bladder cancer patients). This process was repeated for each machine learning algorithm. This resulted in 3 candidate models composed of a standardizer, missing data imputer, and classifier ready to be applied to new data.

Second, model candidates were applied on 20% of CDRD to verify their generalization power. From there, the best classification approach was identified by comparing the performance using AUC, sensitivity, and specificity. At the end of this step, training using all CDRD data, the best hyperparameter set, and the best approach was conducted and resulted in the RFM.

### Image-Based Model

**Figure 3:**
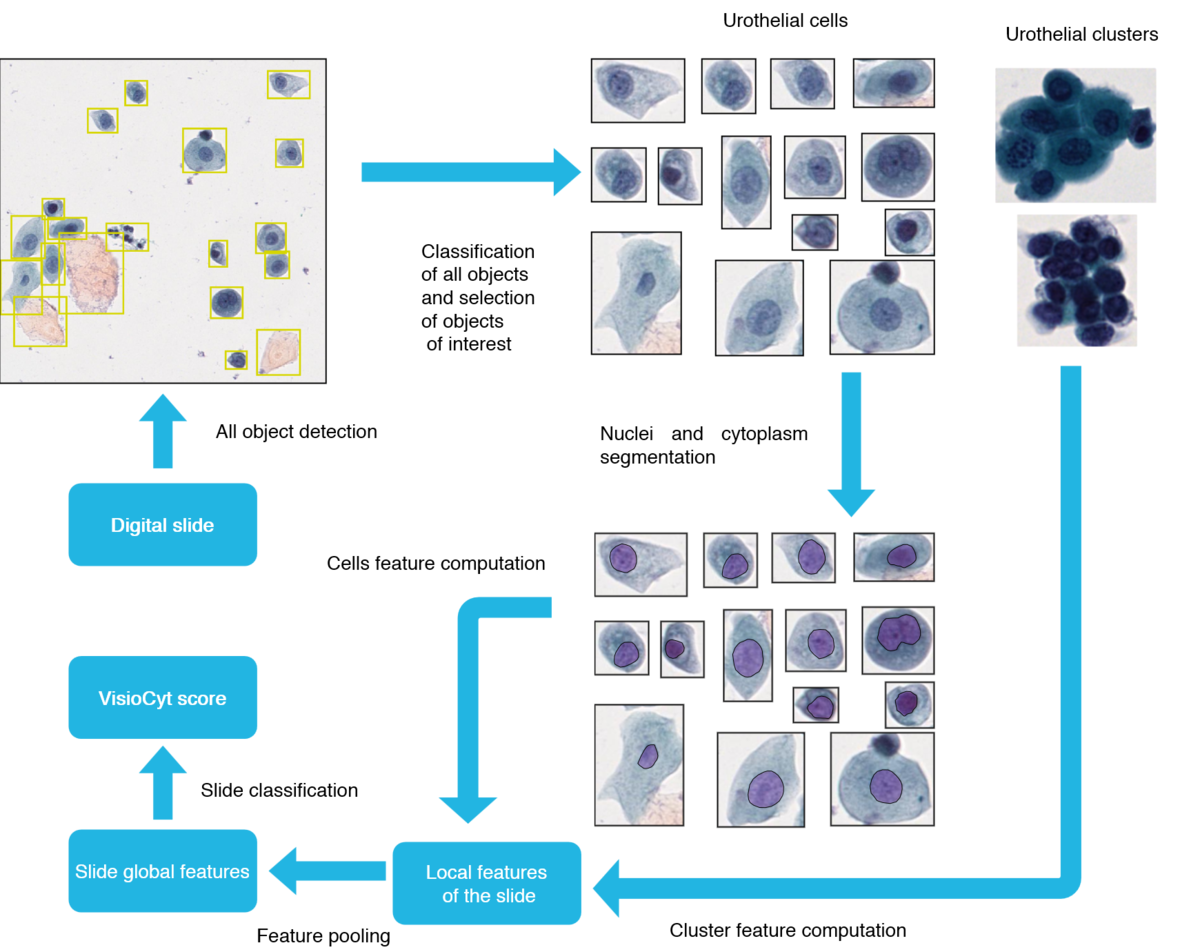
Workflow of the image-based model.

The image-based model works in 5 main steps going from checking the quality of the data to prediction of bladder cancer from segmented cell images. The process is depicted in Figure 3.

The process begins with a quality check of the slide, discarding any that are not properly prepared nor digitized. Following this, the biological elements on the slide are detected and classified, retaining only individual urothelial cells and clusters of these cells. A second quality assessment is then carried out to confirm the presence of a sufficient number of urothelial cells.

These cells are subsequently segmented to identify the nucleus and cytoplasm, and the characteristics of the nucleus are calculated based on established criteria and atypia parameters [23]. Urothelial clusters are classified using a detector for atypical urothelial clusters, and the classification score is used as a feature of the cluster.

Features of both individual urothelial cells and urothelial clusters are combined into a vector representing the features of the slide. This vector is ultimately classified by an RF classifier to determine the VisioCyt slide’s score.

### Combination of Image-Based and Risk Factor–Based Models

At this stage, prediction scores obtained from the RFM and VisioCyt are available. To combine these 2 scores and provide the final combined scoring system, 3 strategies were investigated using the output probabilities of both models as inputs: custom, LR, and decision tree (DT).

Experiments and evaluations were performed using the VTD. At first, stratified random selection, based on grade, was performed to allocate 520 patients to a training set and 131 patients to a test set. The most optimal hyperparameters of LR and DT were then identified using 3-fold cross-validation and a random grid search on the training set as described in the Bladder Cancer Prediction From Clinical Data section. The best models were then applied to the test set. For the custom method, the behaviors of the RFM and VisioCyt scores to build a decision rule adapted to our data were examined on the training set.

VisioCyt and RFM were not trained but simply applied on the training and test sets to compare their performance with those of the combination approaches.

### Statistical Analysis and Evaluation Metrics

The relationships between the clinical variables and target variable in both CDRD and VTD were investigated. First, the correlations between bladder cancer status (ie, positive or negative) and continuous variables were studied using the point-biserial correlation test. According to Vogt et al [26], only features with point biserial values >0.25 were considered significantly correlated with the target variable. Chi-square tests were conducted for categorical features. This test consists of a null hypothesis that supposes the independence between the binary feature and bladder cancer status. Using Bonferroni correction, we considered that a *P*<.003 indicated a rejection of the null hypothesis and therefore implied a correlation between the binary variable and bladder cancer status.

ROC curve and AUC analyses were carried out to evaluate the performance of models. In addition, commonly used performance metrics such as balanced accuracy, specificity, and sensitivity were assessed for the RFM. They were measured using the optimal threshold given by the ROC as the combination maximizing the difference between the true positive and false positive rates. To better understand how the models made the predictions, the importance of the features on the predictions was investigated. Although intrinsically present in RF and DT models [27], this information is not directly available for other approaches such as SVMs. In such cases, the permutation feature importance was computed [27].

### Software and Platforms

All experiments were implemented in Python, version 3.8.8. Statistical analyses were performed using scipy 1.7.3. Scikit-learn 1.0.2 was used to train and evaluate the classification models.

## Results

The results are presented in 3 sections. First, examination of the clinical features was undertaken to assess their univariate predictive capability for bladder cancer and to evaluate their consistency or heterogeneity across the 2 studied data sets. Second, the performances achieved during the design of the RFM are outlined. Last, the results obtained from the combination of the 2 scoring systems are discussed.

### Clinical Feature Analysis

First, the distribution of the clinical features can be discussed by comparing either populations (ie, negatives vs positives) or data sets (ie, CDRD vs VTD). The statistics were grouped within these objectives and are reported in Table 1.

**Table 1:** Distribution of variables across positive and negative bladder cancer status for each data set.

Clinical features			Positive status		Negative status	
			CDRD^a^ (n=1351)	VTB^b^ (n=409)	CDRD^a^ (n=4071)	VTB^b^ (n=211)
**Continuous variables, mean (SD)**						
	Age (years)		69.8 (12.9)	70.7 (10.0)	58.8 (17.7)	63.9 (13.1)
	Weight (kg)		74.6 (13.8)	76.1 (14.9)	73.4 (33.1)	74.6 (16.1)
	Height (cm)		168.2 (7.5)	170.0 (7.1)	167.2 (7.1)	168.0 (10.0)
	BMI (kg/m^2^		26.4 (4.5)	26.0 (4.2)	26.3 (11.6)	26.2 (4.7)
**Binary variables, n (%)**						
	Sex (male)		1069 (79.1)	321 (78.5)	2189 (53.8)	118 (55.9)
	**Smoking status**					
		Never smoked	482 (48.8)	83 (20.5)	641 (17.7)	114 (54.8)
		Active smoker	109 (37.2)	91 (22.5)	384 (25)	27 (13)
		Former smoker	301 (15.4)	231 (57)	201 (6.9)	67 (32.2)
	Diabetes		243 (21.9)	86 (21)	389 (19.3)	19 (9)
	Heart failure		150 (35.1)	91 (22.2)	380 (30.2)	9 (4.3)
	Hypertension		311 (69.1)	210 (51.3)	830 (52.1)	55 (26.1)
	Stroke		87 (16.3)	22 (5.4)	261 (14.7)	1 (0.5)
	COPD^c^		95 (17.9)	83 (20.3)	140 (10.1)	18 (8.5)
	Professional exposure		40 (2.7)	101 (24.7)	80 (1.3)	23 (10.9)
	Family history of cancer		13 (0.9)	67 (16.4)	35 (0.8)	40 (19)

^a^CDRD: Clinical Data Reuse Dataset.

^b^VTD: VisioCyt 1 Trial Dataset.

^c^COPD: chronic obstructive pulmonary disease.

The distributions of continuous features (age, weight, height, and BMI) were equivalent in both data sets and between negatives and positives. For binary features, 3 scenarios emerged: Some display equivalent percentages and trends (ie, sex and COPD), while others only shared similar trends (active smoker, former smoker, heart failure, and hypertension) and others exhibited differences in both percentages and trends (never smoked, diabetes, stroke, professional exposure, and family history of cancer).

For sex and COPD, percentages and trends were similar in CDRD and VTD. There were more men with bladder cancer than without (with cancer: 1069/1351, 79.1% in CDRD and 321/409, 78.5% in VTD; without cancer: 2189/4071, 53.8% in CDRD and 118/211, 55.9% in VTD), and there was a higher occurrence of COPD among individuals with bladder cancer (95/1351, 17.9% in CDRD and 83/409, 20.3% in VTD) than in individuals without bladder cancer (140/4071, 3.4% in CDRD and 18/439, 8.5% in VTD).

For features with only similar trends, such as being an active smoker, heart failure, and hypertension, the percentages were generally higher in CDRD than in VTD, except for being a former smoker, where lower percentages were observed in CDRD than in VTD (Table 1).

For features considered to have different percentages and trends (never smoked, diabetes, stroke, professional exposure, and family history of cancer), the key observation was the equivalence between positives and negatives in CDRD, while a more pronounced difference existed between positives and negatives in VTD.

Statistical analyses were performed to observe relationships between features and bladder cancer status in each data set. The results are reported in Table 2.

**Table 2:** Statistical analyses performed to evaluate the correlations between the clinical features and bladder cancer status for each data set.

Clinical features			Clinical Data Reuse Dataset (CDRD)		VisioCyt 1 Trial Dataset (VTD)	
			Statistic	*P* value	Statistic	*P* value
**Continuous variables, r_pb_^a^**						
	Age		0.276	<.001	0.276	<.001
	Weight		0.018	.18	0.051	<.001
	Height		0.059	<.001	0.133	<.001
	BMI		0.004	.74	–0.025	.54
**Binary variables, χ^2^ (*df*)^b^**						
	Sex		108.534 (1)	<.001	10.005 (1)	<.001
	**Smoking status**					
		Never smoked	0.659 (1)	.42	49.854 (1)	<.001
		Active smoker	53.680 (1)	<.001	6.535 (1)	<.001
		Former smoker	81.775 (1)	<.001	17.705 (1)	<.001
	Diabetes		3.375 (1)	.04	11.879 (1)	<.001
	Heart failure		7.824 (1)	<.001	27.911 (1)	<.001
	Hypertension		52.403 (1)	<.001	20.809 (1)	<.001
	Stroke		1.710 (1)	.17	9.027 (1)	<.001
	COPD^c^		51.868 (1)	<.001	11.822 (1)	<.001
	Professional exposure		13.198 (1)	<.001	13.242 (1)	<.001
	Family history of cancer		0.073 (1)	.92	0.535 (1)	.46

^a^Values >0.25 considered significant.

^b^*P* values <.003 considered significant.

^c^COPD: chronic obstructive pulmonary disease.

For 8 features (ie, age, sex, active smoker, former smoker, heart failure, hypertension, COPD, and professional exposure)*,* relationships with bladder cancer were highlighted in both CDRD and VTD, while 3 others were only considered related to bladder in VTD (diabetes, never smoked, and stroke). Finally, 4 features (weight, height, BMI, and family history of cancer) were not considered significant in any of the data sets.

### Bladder Cancer Prediction Using Clinical Features

This section is dedicated to the investigations of the RFM. First, the best hyperparameters for each classification approach are reported. The performance between model candidates is then compared. To finish, the importance of each feature in the best model is investigated.

#### Hyperparameter Tuning

During the training phase, hyperparameters were tuned for each of the 3 classification approaches (RF, SVM, LR; Table 3).

In the end, the RF model candidate was composed of 45 trees with a maximum depth of 10 splits, minimum sample split of 10, and minimum sample leaf at 30 samples each. The RF model had an average AUC of 0.76 (SD 0.02). The SVM candidate was a linear kernel with a margin at 10 and 0.01 for gamma and had an average AUC of 0.77 (SD 0.02). The LR candidate embedded a margin at 0.01 with an l1 penalty and performed with an average AUC of 0.77 (SD 0.02). Model candidates were finally trained using these hyperparameters on the CDRD training set.

**Table 3:** Summary of the tuning of the hyperparameters.

Classifier and tuned hyperparameters		Tested values	Best AUC^a^, mean (SD)
**Random forest**			0.76 (0.02)
	number of trees	(10, 14, 18, 23, 27, 32, 41, 45^b^, 50)	
	maximal depth	(5, 6, 7, 8, 10^b^, 13, 20)	
	minimal sample split	(5, 10^b^)	
	minimal sample leaf	(4, 10, 30^b^, 50)	
**Support vector machine**			0.77 (0.02)
	kernel	(linear^b^, radial basis function)	
	margin	(0.001, 0.01, 0.1, 1, 10^b^, 100)	
	gamma	(0.001, 0.01^b^, 0.1, 1, 10, 100)	
**Logistic regression**			0.77 (0.02)
	margin	(0.001, 0.01^b^, 0.1, 1, 10, 100)	
	penalty	(l1^b^, l2, none)	

#### Performance Comparison

**Figure 4:**
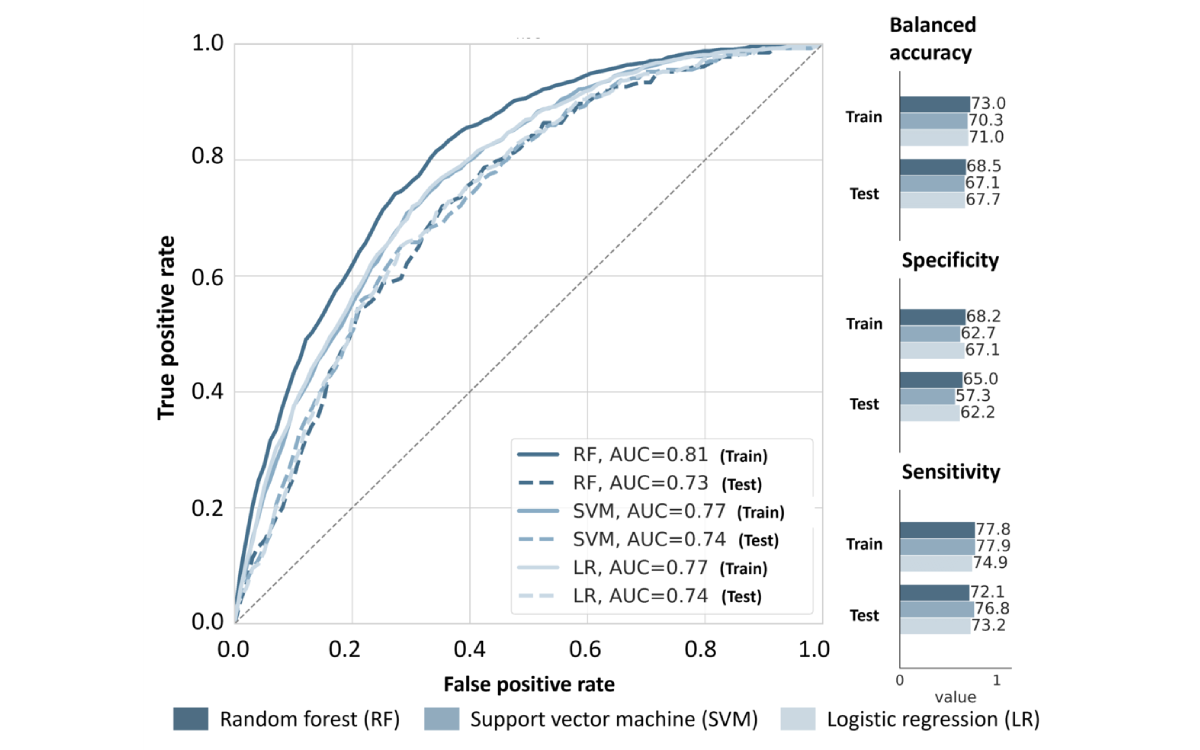
Receiver operating characteristic (ROC) curve comparing the performance of risk factor–based classifiers in terms of the area under the ROC curve (AUC), balanced accuracy, specificity, and sensitivity for both the training and test sets.

To identify the best candidate to be deployed, performance on the training set (80% of CDRD) and applied once to the test set (20% of CDRD) were compared for each modeling approach. The resulting performances are reported in Figure 4.

When looking at the AUC, all approaches succeeded, with values above 0.73 on the test set. However, there was a stronger overfitting with the RF candidate (ie, a drop of 0.08 between the training and test sets was observed). The other 2 models had similar AUCs on the training and test sets: 0.77 for SVM and 0.74 for LR. This indicates that these models generalized the problem better. Regarding the other evaluation metrics, the LR candidate had a better sensitivity-specificity balance (ie, sensitivity of 73.2% and specificity of 62.2% on the test set) than the SVM (ie, sensitivity of 76.8% and specificity of 57.3% on the test set). In the end, the LR candidate was chosen for further investigation as the RFM (ie, feature importance analysis and combination with VisioCyt).

#### Impact of Clinical Features on Predictions

The individual importance of each feature for the prediction with LR is reported in Table 4.

**Table 4:** Feature importance of the risk factor–based model.

Feature	Importance model
age	0.14
sex	0.05
BMI_was_missing	0.03
smoking_status_active	0.03
smoking_status_former	0.01
heart_failure	0.01
weight	0.01
BMI	0.01
COPD	<0.01
stroke	<0.01
smoking_status_never	<0.01
professional_expo	<0.01
diabetes	<0.01
height	<0.01
COPD_was_missing	<0.01
hypertension_was_missing	<0.01
heart_failure_was_missing	<0.01
stroke_was_missing	<0.01
professional_expo_was_missing	<0.01
height_was_missing	<0.01
smoking_status_active_was_missing	<0.01
tumor_family_was_missing	<0.01
smoking_status_former_was_missing	<0.01
weight_was_missing	<0.01
sex_was_missing	<0.01
diabetes_was_missing	<0.01
smoking_status_never_was_missing	<0.01
tumor_family	<0.01
hypertension	<0.01

Unsurprisingly, age and sex were associated with the highest importance values (0.14 and 0.05, respectively). Information on patients’ smoking status, through *smoking_status_active* and *smoking_status_former* features, had an impact on the predictions. In addition, weight and BMI were taken into consideration along with information on the presence or absence of BMI features (*BMI_was_missing*). Finally, the impact of a history of heart attacks (*heart_failure*) was also considered by the model. All other features had negligible univariate significance.

**Figure 5:**
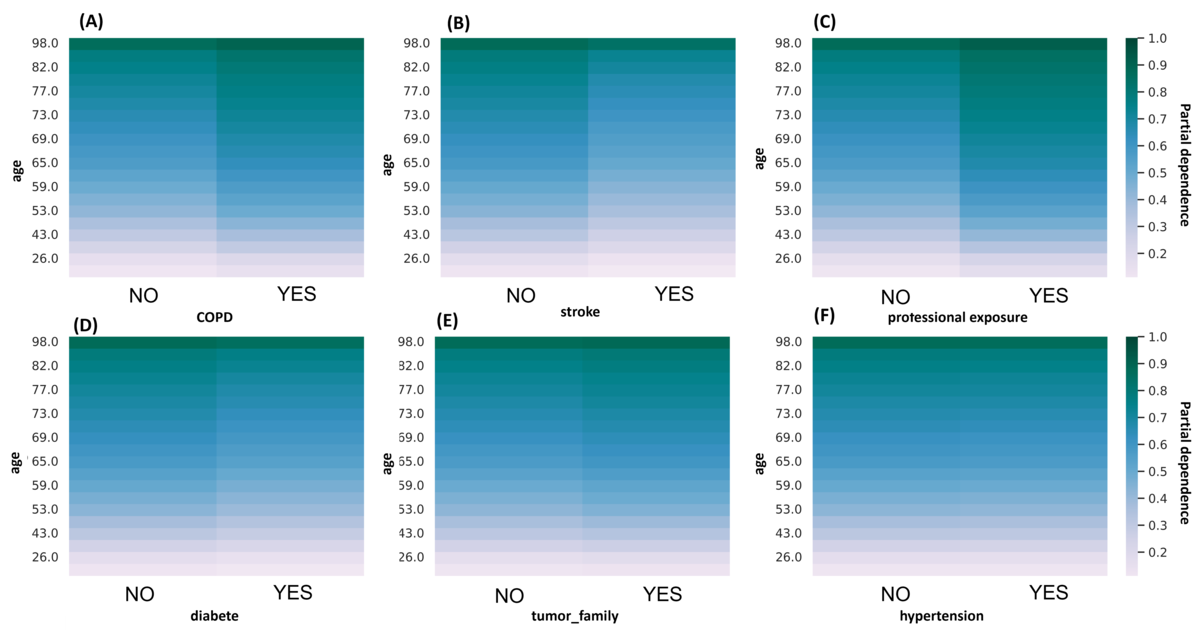
Partial dependence plots for 6 feature combinations of age with (A) chronic obstructive pulmonary disorder (COPD), (B) stroke, (C) professional exposure, (D) diabetes, (E) tumor family, and (F) hypertension.

However, we could assume that the associations between these variables are the important factor in the model rather than when they are considered 1 by 1. Looking at 2-feature partial dependence plots allows us to explore this hypothesis. The combinations of age with features of low importance were investigated (ie, COPD, stroke, *professional_expo*, diabetes, *tumor_family*, and hypertension) and are presented in Figure 5.

Patient age played an important role. Indeed, the highest probabilities (in dark green) were observed in the oldest patients. Higher risks (darker areas on the right than on the left of figures) were associated with COPD, professional exposure, and a family history of bladder cancer (*tumor_family*). This suggests that the model successfully captured the effects associated with these risk factors commonly identified in the literature. A history of stroke and the presence of diabetes were also considered but with the opposite effect than expected (absence increases risk). No impact of the hypertension feature was observed here.

### Bladder Cancer Prediction by Combining RFM and VisioCyt

#### Building the Custom Method

**Figure 6:**
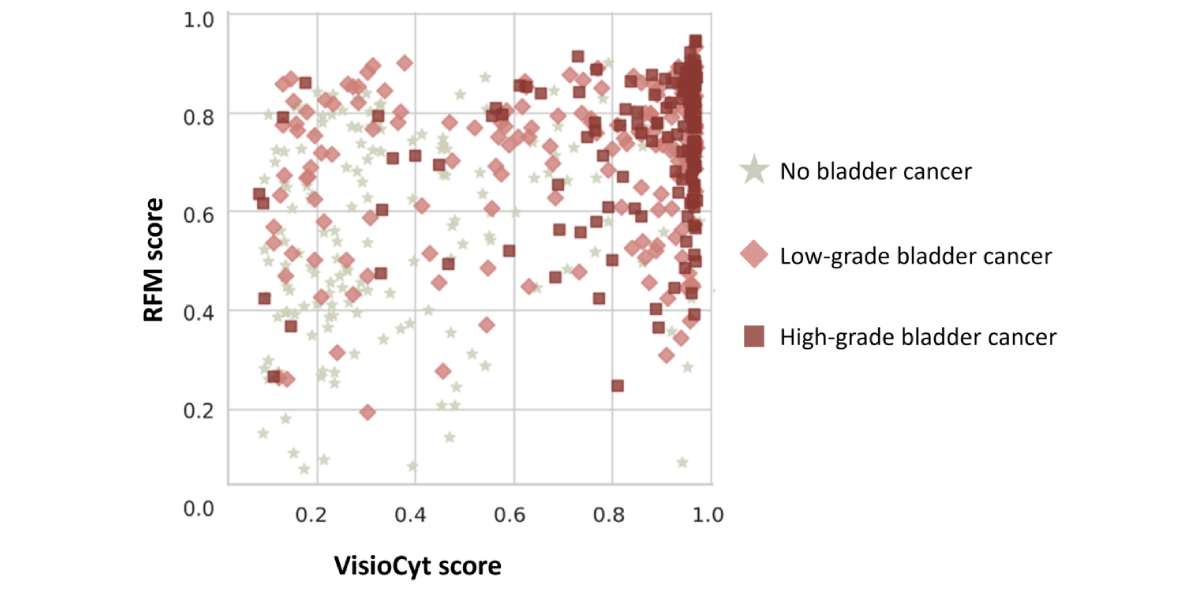
2D plot of risk factor–based model (RFM) and VisioCyt scores on the VisioCyt 1 Trial Dataset (VTD) training set.

The resulting scores for the VTD training set are reported in Figure 6.

The first possible observation was that the detection of high-grade cancer was broadly identifiable by the high values of both scores. A VisioCyt score >0.6 indicates a high probability of cancer. In fact, very few patients without bladder cancer were in this zone of the scatter plot. Below this threshold, detection of low-grade cancers seems more difficult using the insight from VisioCyt alone. However, the RFM score was often higher than 0.6 for these patients. From these observations, the custom combination rule was defined as follows:

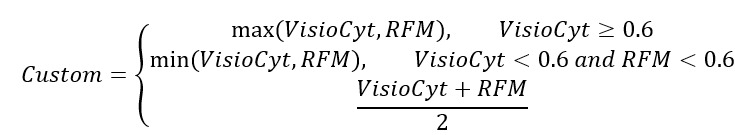



#### Identification of the Best Modeling Approach

At first, the LR and DT models were subjected to 3-fold cross-validation on the training set to identify the best hyperparameters. LR with l1 regularization and a margin of 78.8 led to an AUC of 0.81 (SD 0.02) on the validation sets. DT with a minimum sample leaf of 5, maximum depth of 2, and a *Gini* criterion resulted in an AUC of 0.78 (SD 0.02). These hyperparameters were used to retrain the 2 approaches on the VTD training set.

**Figure 7:**
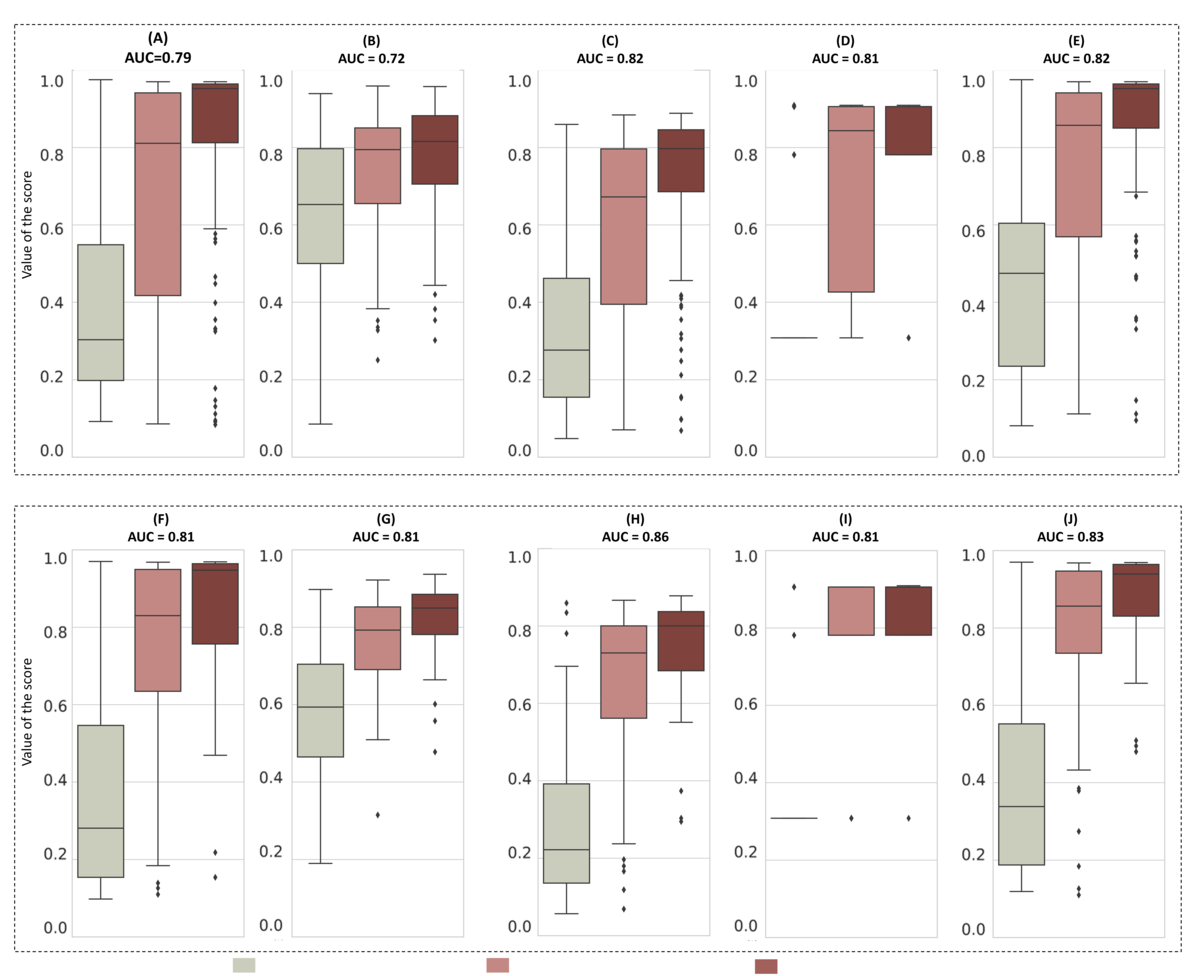
Distribution of the scores by bladder cancer grade for the VisioCyt 1 Trial Dataset (VTD) training set using (A) VisioCyt, (B) the risk factor–based model (RFM), (C) logistic regression (LR), (D) decision tree (DT), and (E) a custom strategy and for the VTD test set using (F) VisioCyt, (G) RFM, (H) LR, (I) DT, and (J) a custom strategy.

The comparison between the 5 bladder cancer predictors is illustrated in Figure 7. It compiles the AUCs and score distributions according to bladder cancer severities of the image-based model, the RFM, and the 3 strategies for score combination: LR, DT, and custom. Initial scores obtained with VisioCyt and RFM are reported on the left, while scores from the combination using LR, DT, and custom lie on the right. Values obtained on the training and test sets of VTD were included to discuss the generalization of both approaches.

The first element to be noted is that combinations of the image-based model and RFM resulted in better performances in terms of AUC independent of the combination strategy (LR, DT, or custom). Indeed, the AUCs were higher for the training set (0.82 for LR and custom and 0.81 for DT vs 0.79 for VisioCyt and 0.7 for RFM) and higher or equivalent on the test set (0.86 for LR, 0.81 for DT, and 0.83 for custom vs 0.81 for VisioCyt and RFM). In the light of these sole observations, it was difficult to define the most relevant approach between LR, DT, and custom. To explore this further, we proposed studying boxplots of the VisioCyt, CBM, DT, and custom scores on the VTD training and test sets.

The median values of the scores obtained with LR were distinct for the 3 groups: (1) for patients without bladder cancer: 0.28 on the training set and 0.23 on the test set; (2) for patients with low-grade cancer: 0.68 on the training set and 0.72 on the test set; (3) for patients with high-grade cancer: 0.80 on the training set and 0.81 on the test set. The positive effect of this combination was a slight reduction in the overlap of scores between low-grade patients and those without cancer observed with VisioCyt. However, the IQRs remained quite wide.

The median DT scores showed that DT scores were well separated for patients without (0.30) and with bladder cancer (0.84 for low-grade cancer and 0.78 for high-grade cancer). At first glance, these scores appeared to be more discriminating than the VisioCyt score alone. However, on the test set, IQRs of the DT scores were null for no bladder cancer and equivalent for low-grade and high-grade cancers, which does not allow for continuity in the measurements. This behavior does not allow for accurate interpretation. The doubt about this behavior was confirmed by the fact that the DT scores obtained for these patients during training resulted in a high IQR of 0.42, making it difficult to be sure that patients with low-grade cancer will be correctly associated with a high score. This edge effect also arose because a tree depth of 2 was selected during hyperparameter tuning, limiting the model’s capacity to create more granular splits and accurately differentiate between subgroups in the population.

Regarding the custom approach, the scores associated with low-grade cancer (0.86 on the training set and 0.86 on the test set) and high-grade cancer (0.95 on both sets) were higher than the scores for patients without bladder cancer (0.47 on the training set and 0.34 on the test set). The range of scores for high-grade cancer was narrower with custom than with VisioCyt, indicating that several scores were improved and increased. Regarding patients with low-grade cancer, the median scores were higher with custom (eg, 0.89 on the test set) than with VisioCyt (eg, 0.83 on the test set). In addition, on the test and training sets, the overlap between scores for no bladder cancer patients and for patients with low-grade cancer was weaker than for VisioCyt scores for no cancer. The latter scores were also higher but remained distinct from those of the other 2 categories.

**Figure 8:**
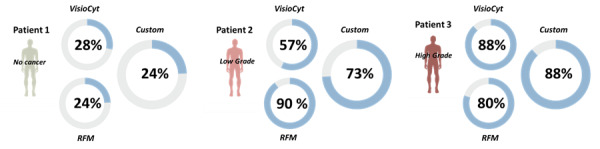
Illustration of the scores given by VisioCyt, the risk factor–based model (RFM), and the custom model for 3 patients in the VisioCyt 1 Trial Dataset (VTD) test set.

In light of these observations, the custom combination seems to be the most effective approach. The 95% CIs for the output scores were 0.33 to 0.52 for no bladder cancer, 0.68 to 0.83 for low-grade cancer, and 0.8 to 0.91 for high-grade cancer in the test set. The main advantage of the custom combination was improved detection of patients with low-grade cancer and increased certainty of the presence of high-grade cancer. One patient in each category was randomly drawn from the test set to illustrate what may be provided to clinician decision-making (Figure 8).

For patient 1, who did not have bladder cancer, the 2 initial scores were quite equivalent: 28% for VisioCyt and 24% for RFM. This also resulted in a low combined risk (28%). For patient 2, who had low-grade bladder cancer, the Visiocyt score was 57%, and the RFM score was 90%, giving a combined score of 73%. For patient 3, who had high-grade bladder cancer, the VisioCyt score was 88%, and the RFM score was 80%, leading to a combined score of 88%. The behaviors of the scores for these 3 patients were well in line with their actual diagnoses.

Although our goal was to create a decision support tool, not a bladder cancer detector, we evaluated the sensitivity and specificity by evaluating the thresholds of the scores obtained with the custom approach. The optimal cut-off point, which maximized the difference between true and false positive rates, was identified through ROC curve analysis. This yielded a threshold of 0.61 which results in 81% sensitivity and 73% specificity on the training set and 76% sensitivity and 83% specificity on the test set.

## Discussion

### Principal Findings

In this study, our team aimed to reuse large-scale clinical data to improve the performance of an image-based model to predict bladder cancer.

As a first step, an RFM was developed using clinical features from 5422 patients whose data were extracted from the CDW. Clinical features were selected because they were identified as important risk factors in the literature. The best RFM, based on LR, succeeded, with an AUC of 0.74, at classifying patients with or without bladder cancer from clinical features. The most predictive factors were age, sex, and smoking status (active or former). We also saw that risk factors such as COPD, professional exposure, and family history of bladder cancer, when combined with age, impacted the model’s predictions. This model is simple and easily interpretable, successfully passing both internal (mean AUC 0.72, SD 0.02 during cross-validation and 0.74 on the CDRD test set) and external (AUC 0.81 on the VTD test set) validation tests.

In the second step, several combinations of this model with an image-based model were proposed. Classical approaches based on LR and DT were proposed and supplemented by a custom approach grounded in RFM and VisioCyt score interpretation. We saw that all combinations gave a better AUC than RFM or VisioCyt alone. However, to identify the best combination, we stratified the performance by grade and chose the one that improved low-grade bladder cancer detection. The custom method (with an AUC of 0.83 on the VTD test set) provided the best results by assigning a median score of 0.34 to patients with no cancer, 0.86 to patients with low-grade cancer, and 0.95 to patients with high-grade cancer. Finally, we proposed observing the VisioCyt, RFM, and custom scores for 3 randomly selected patients, 1 from each of the 3 categories.

The resulting scores are supplementary and hold potential for enhancing the decision-making process. Indeed, they offer valuable insight into the patient's clinical profile, further improving image-based tools. Although the AUC bears resemblance to the figures observed in the studies conducted by Sanghvi et al [14] and Awan et al [15] (ie, 0.80 and 0.81, respectively), direct comparison was not feasible due to the focus on patients with high-grade cancer in those studies, which did not encompass an evaluation of performance for patients with low-grade cancer. Nevertheless, it is essential to underscore that the RFM is not designed for competitive purposes but rather to serve as a complementary component to these tools, much in the same manner as it did with VisioCyt. Combining RFM with these models may result in an even more robust composite score.

These results show the value of reusing data to propose better bladder cancer scoring strategies. Indeed, the strength of learning on thousands of patients has allowed us to propose a model that enhances the scoring by an image-based model for more accurate identification of patients with low-grade cancer.

### Limitations

Some limitations of this work should be noted. First, the multiple missing values during clinical feature extraction raise concerns about the quality of the process. Multimedia Appendix 1 provides insight on the availability of each feature before imputation. This is an inherent problem in data reuse, which is not the primary purpose of the collection, because the information is not collected by default. Indeed, we are uncertain if these concepts are consistently present in all patient documents, as some data are recorded subjectively by clinicians. This is particularly true for variables such as COPD or a family history of cancer, features for which our data collection was limited in this study.

However, sometimes the information may still be hidden in unstructured data, which we are unable to exploit today. We used relatively straightforward extraction methods, possibly resulting in incomplete information retrieval from the reports. To address this, we plan to explore more advanced natural language processing methods like bilateral long short-term memory models or large language models [28], but their implementation and evaluation will be challenging due to limited annotations of the specific clinical concepts we sought to extract and the criticality of the confidentiality of electronic health records.

This last point could explain why we obtained feature distributions in CDRD that were sometimes different from those in VTD. However, they also may be explained by the fact that CDRD is larger than VTD since it includes a broader population. In fact, the percentage of patients with cancer was higher in VTD (66%) than in CDRD (25%). Overall, both data sets had the same characteristics, and most of the features were significant for bladder cancer detection. Some differences were nevertheless observed, and we will have to remain vigilant on these variables (ie, never smoked, stroke). We also observed the opposite behavior to what might be expected for diabetes and stroke in the RFM. Hypertension, though recognized as important in the literature, was not considered by the model. These are elements that deserve to be studied in greater detail once extraction methods are more robust.

Moreover, only 29 features were used, and they were often binary. Obtaining more complete information by either adding other clinical features (ie, biological measurement) or using a finer scale (ie, the type of diabetes or the number of cigarettes smoked per day) is an improvement not to be neglected and may increase the specificity of RFM. The same strategy can be used to refine the target. Indeed, it may be relevant to classify patients according to grade rather than in a binary way. Similarly, the proposed model was only based on features that we found to be linked with bladder cancer in the literature, but it will be interesting to add other comorbidities that may be cofounding factors (such as having another type of cancer).

In addition, we observed that the custom approach worked best. Within one of the defined score intervals, a simple average of the 2 scores was performed. A weighting may be integrated to fuse scores through an optimal fusion strategy [29].

Finally, although we carefully evaluated our models with regards to our available data sets, a concern for generalization may be observed later since only 1 data set with both cytology and clinical data (VTD) was investigated. First, we expect to enrich our RFM model by working on all the Hugo network’s health data warehouses [24]. We also intend to deploy the combined model on new data to verify its stability and ensure relevance in routine care. Indeed, at the moment, the proposed combination has not yet been tested and approved by clinicians. However, it is important to consider that the collection of cytology images associated with clinical data requires the implementation of new acquisition protocols and that it will require time.

### Conclusions

The results of this study are encouraging. This is the first time that the contribution of a prediction model based on risk factors identified in the literature and on the reuse of massive data has been studied. We observed that it can reinforce recent tools such as urine cytology–based models. Specifically, it may improve low-grade bladder cancer detection.

## Appendix

Multimedia Appendix 1 Values, type and interpretation of each feature and percentage of missing values across each feature in the Clinical Dataset (CDRD) and the VisioCyt 1 Trial Dataset (VTD) before imputation.

## Abbreviations

AUC: area under the receiver operating characteristic curve
CDRD: Clinical Data Reuse Dataset
CDW: clinical data warehouse
COPD: chronic obstructive pulmonary disease
DT: decision tree
ICD-10: International Statistical Classification of Diseases, Tenth Revision
LR: logistic regression
PMSI: Programme de Médicalisation des Systèmes d'Information
RF: random forest
RFM: risk factor–based model
ROC: receiver operating characteristic
SVM: support vector machine
VTD: VisioCyt 1 Trial Dataset

## Data Availability

The clinicobiological data used in this work contain pseudoidentifiable information and cannot be shared directly. However, a collaboration with the Clinical Data Center of Rennes Hospital can be considered to allow the use of these data while adhering to legal and regulatory frameworks. Please contact the corresponding author for more information. However, the conditions under which cytology data were collected do not permit their sharing.
