# Supplementary figures and images for: Combining a Risk Factor Score Designed From Electronic Health Records With a Digital Cytology Image Scoring System to Improve Bladder Cancer Detection: Proof-of-Concept Study
Source: J Med Internet Res. 2025 Jan 22;27:e56946. doi: 10.2196/56946 (PMC11799811; doi:10.2196/56946)

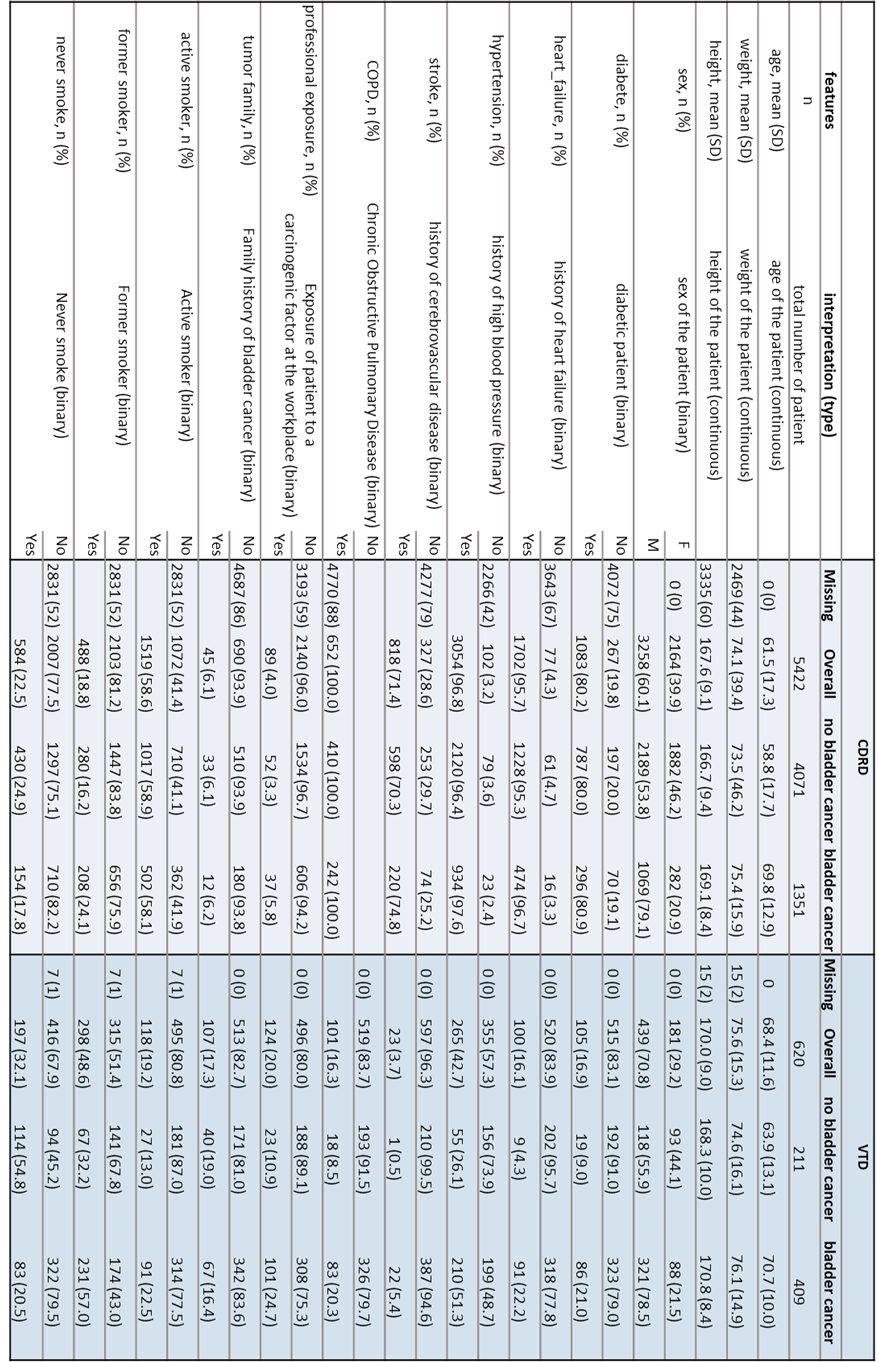

Supplement: Multimedia Appendix 1 [file jmir_v27i1e56946_app1.docx]
